# Enhancing OLED Performance by Optimizing the Hole Transport Layer with a Self-Assembled Monolayer

**DOI:** 10.3390/ma18040748

**Published:** 2025-02-08

**Authors:** Ziying Niu, Yongqiang Wang, Zhenjiang Xu, Yunlong Liu, Wenjun Wang, Shuhong Li

**Affiliations:** School of Physical Science and Information Technology, Liaocheng University, Liaocheng 252059, China; n18536136362@163.com (Z.N.); wyq2385993945@163.com (Y.W.); 17661822959@163.com (Z.X.); liuyunlong@lcu.edu.cn (Y.L.)

**Keywords:** molecular transition dipole moment, organic light-emitting diode, self-assembled monolayer

## Abstract

The enhancement of organic light-emitting diode (OLED) device performance has been a key area of research in organic optoelectronic devices. Optimizing carrier mobility within OLED devices is a crucial strategy. In this study, the hole transport layer was optimized using a self-assembled monolayer (SAM) subjected to different annealing temperatures. Through submitting the SAM to an annealing temperature of 100 °C, a maximum luminous intensity of 32,290 cd/m^2^ and a maximum EQE of 1.77 were achieved, the latter being more than two-fold higher than that without the SAM. As the SAM annealing temperature increased from 80 °C to 120 °C, both the vertical orientation of molecules in the hole transport layer and the hole mobility in hole-only devices (HODs) improved. This vertical orientation is beneficial to enhancing hole mobility. Electrochemical impedance spectroscopy and surface morphology analysis revealed that the introduction of a SAM leads to the formation of interface resistance. The synergy effect between the variation in the molecular transition dipole moment and the interface morphology of the hole transport layer optimizes the hole mobility of HODs and leads to the enhancement of OLED performance.

## 1. Introduction

Since Ching W. Tang reported the world’s first organic light-emitting diode (OLED) in 1987, OLED devices have been widely used in the display and lighting fields for their advantages of low power consumption, high brightness, wide spectrum, excellent color rendering, and high efficiency [1,2,3]. To improve the mismatch between the low work function of indium tin oxide (ITO) and the highest occupied molecular orbital (HOMO) energy level of the hole transport material, various approaches, including hole injection layer insertion, buffer layer insertion, and self-assembly monolayer (SAM) methods, have been explored to address this issue [4].

SAMs are organized molecular structures created through the adsorption of active surfactants on solid surfaces [5]. Due to its simplicity in preparation, good stability, and diverse testing methods, SAM technology is garnering increasing attention and significance in the fields of optoelectronic conversion devices and electrochemistry [6]. Shuo Li et al. achieved optimized pentacene thin-film transistors with enhanced mobility (up to 0.68 cm^2^ V^−1^ s^−1^) and on/off ratios (>10^6^) through SAM treatments, demonstrating the critical role of interface charge transport control in nanoscale materials [7]. Organic solar cells with a ZnO electron transport layer modified by SAMs or hole transport SAM layers demonstrated a remarkable improvement in short-circuit current density and overall efficiency [8,9,10,11,12,13,14]. Fei Huang et al. proposed a strategy to achieve hole injection layer (HIL)/hole transport layer(HTL)-free OLEDs by modifying ITO, with SAMs of various phosphoric acids demonstrating the most effective hole injection performance [15]. Myung-Gyun Baek et al. investigated the effects of using SAMs with different functional groups as hole injection layers on OLED device performance [16]. Deng et al. enhanced OLED hole injection efficiency and stability by modifying the ITO interface with 3-aminobenzeneboronic acid (3ABBA), thereby reducing the hole injection barrier and device hydrophilicity [17].

In this paper, a solution-processed SAM was optimized under various annealing temperatures. At an annealing temperature of 100 °C, the luminous intensity of the OLED increased to more than double that of the OLED without SAM. The synergistic effect between the variation in the molecular transition dipole moment (TDM) and the interface morphology of the HTL optimized hole mobility, resulting in the enhanced performance of the OLED.

## 2. Experimental Section

SAM (4-(3,6-dimethyl-9H-carbazole-9-yl) butyl) phosphonic acid (Me-4PACz) was sourced from Jiangsu Aikon Biomedical R&D Co., Ltd. (Nanjing, China). The hole transport layer (HTL) of N, N′-diphenyl-N, N′-(1-naphthyl)-1,1′-biphenyl-4,4′-diamine (NPB) and the emission layer (EML) of tris(8-hydroxyquinoline) aluminum (Alq3) were obtained from Xi’an Bath Sun Technology Co., Ltd. (Xi’an, China)

ITO glass substrates with a square resistance of 15 Ω/sq, purchased from South China Science & Technology Company Ltd., (Shenzhen, China), were employed. These substrates underwent oxygen plasma treatment for 10 minutes to enhance their functionality and hydrophilicity [18,19,20]. The SAM was prepared via spin coating, while other organic layers were thermally vacuum-deposited. The thickness of all samples was measured by ellipsometry (VASE; M-2000, J.A. Woollam, Lincoln, NE, USA). The TDM of NPB was measured using an angle-dependent fluorescence spectroscopy system produced by Hamamatsu Photonics (Hamamatsu, Japan). Device performance was evaluated using Keithley 2400 from Keithley Instruments, Inc., USA (Beaverton, OR, USA) and Spectra Scan PR655 from Photo Research, Inc., USA (North Syracuse, Rochester, NY, USA), respectively. All OLED device performance tests were conducted in the dark and under unencapsulated conditions.

## 3. Results and Discussion

**Figure 1:**
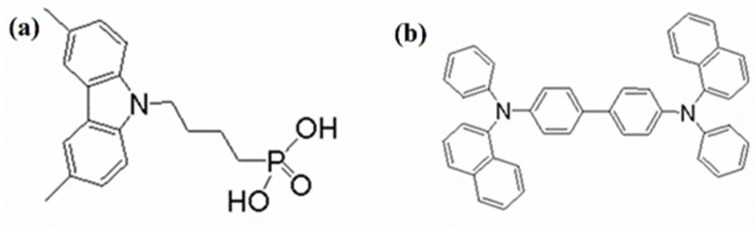
Chemical structure of (**a**) Me-4PACz and (**b**) NPB.

Me-4PACz, known for its self-assembling properties, was utilized in this work to induce the TDM of NPB. Figure 1 gives the chemical structure of Me-4-PACz.

**Figure 2:**
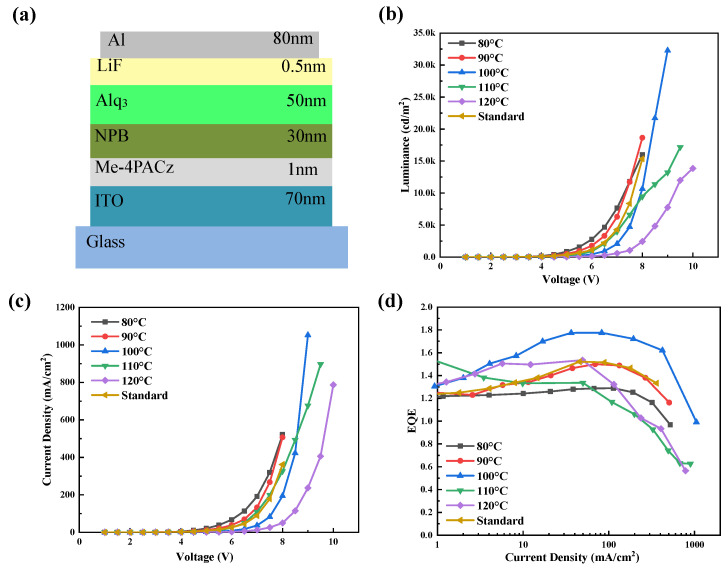
(**a**) OLED device structure. OLED device performance: (**b**) luminance–voltage curves, (**c**) current density–voltage curves (J-V), and (**d**) EQE curves at different annealing temperatures.

A 1 mg/mL chloroform solution of Me-4PACz was prepared and spin-coated onto an ITO substrate at 5000 rpm to form a 1 nm thin film [21]. The films were then annealed on hotplates at 80 °C, 90 °C, 100 °C, 110 °C, and 120 °C for 10 min each. Operating under a vacuum of 5 × 10^−4^ Pa, the vacuum deposition system was utilized to deposit NPB and Alq_3_ at rates of 0.2–0.3 Å/s, LiF at 0.1 Å/s, and Al at 1 Å/s. Molecular self-assembly is a spontaneous process influenced significantly by temperature changes in thermodynamic equilibrium or quasi-equilibrium. Here, the impact of annealing temperature on the molecular self-assembly characteristics of Me-4PACAz was explored and optimized. The structure of the corresponding OLEDs was ITO/Me-4PACz (1 nm)/NPB (30 nm)/Alq3 (50 nm)/LiF (0.5 nm)/Al (80 nm), and the optoelectronic properties were evaluated. Figure 2 shows the current density–voltage–luminance (J-V-L) and EQE curves of OLED devices corresponding to SAMs treated at different annealing temperatures. The “Standard” group serves as a control, referring to OLED devices without a SAM. With the annealing temperature rising from 80 to 120 °C, the maximum luminance and EQE increased first and then declined. At an annealing temperature of 100 °C, the device achieved a maximum luminance of 32,290 cd/m^2^ and a maximum EQE of 1.77%, representing significant improvements of approximately 110% and 16.4%, respectively, compared to the reference device’s maximum luminance of 15,350 cd/m^2^ and maximum EQE of 1.53%. The combined HTL composed of the SAM layer and the NPB layer was beneficial in enhancing the performance of OLEDs. To investigate the chemico-physical mechanism of this phenomenon, the surface morphologies of NPB films with/without SAM layers, the TDM orientation of NPB molecules, and the electrical properties of the hole-only devices (HODs) were studied.

**Figure 3:**
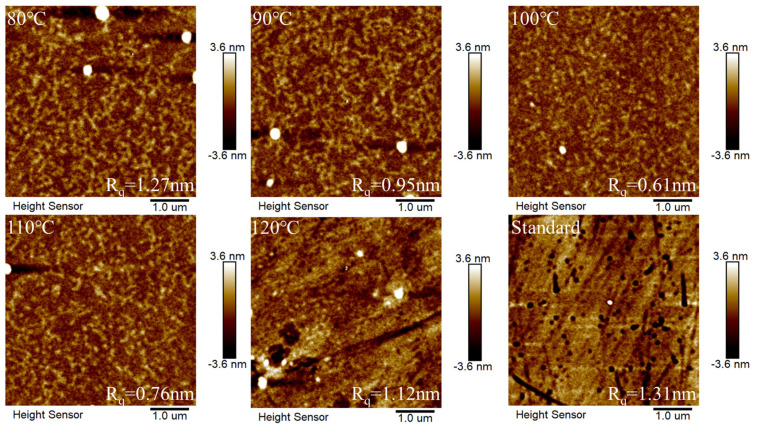
AFM images at different annealing temperatures.

The surface morphologies of NPB films deposited on the Me-4PACz SAM annealed at different temperatures were examined, and the AFM images are shown in Figure 3. The “Standard” serves as a control, referring to NPB films without Me-4PACz. All films exhibit a low root mean square roughness (R_q_). As the annealing temperature rises from 80 °C to 100 °C, the film’s R_q_ progressively diminishes. Conversely, as the annealing temperature increases from 100 °C to 120 °C, the R_q_ of the film gradually increases. The lowest R_q_ was obtained at an annealing temperature of 100 °C.

**Figure 4:**
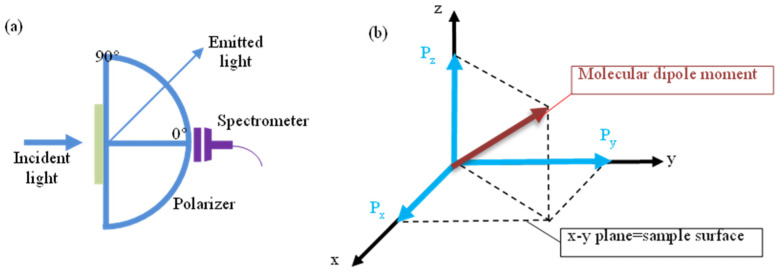
(**a**) Schematic diagram of the angle-dependent fluorescence spectroscopy test device. (**b**) Definition of molecular dipole moment.

Variations in the annealing temperature of SAMs also impact the TDM orientation of the NPB molecule. An NPB film deposited on a blank substrate served as the reference sample. Angle-dependent fluorescence spectroscopy was used to investigate the TDM orientation of NPB films under various conditions [22,23,24]. A schematic diagram for measuring the TDM orientation is shown in Figure 4. A Cartesian coordinate system is established, with the x-y plane as the plane of the film and the *z*-axis as the normal to the film, representing the thickness direction. A material with a dipole orientation can be viewed as a superposition of $p_{x}$, $p_{y}$, and $p_{z}$ dipoles. When the molecular dipole moments are oriented horizontally, the TDMs lie within the x-y plane, parallel to the film surface. Conversely, when the TDM orientation is vertical, which is perpendicular to the film surface, the vertical dipole moment orientation can be expressed as $\Theta_{v}=p_{⊥}/\left(p_{∥}+p_{⊥}\right)=p_{z}/\left(p_{x}+p_{y}+p_{z}\right)$.

**Figure 5:**
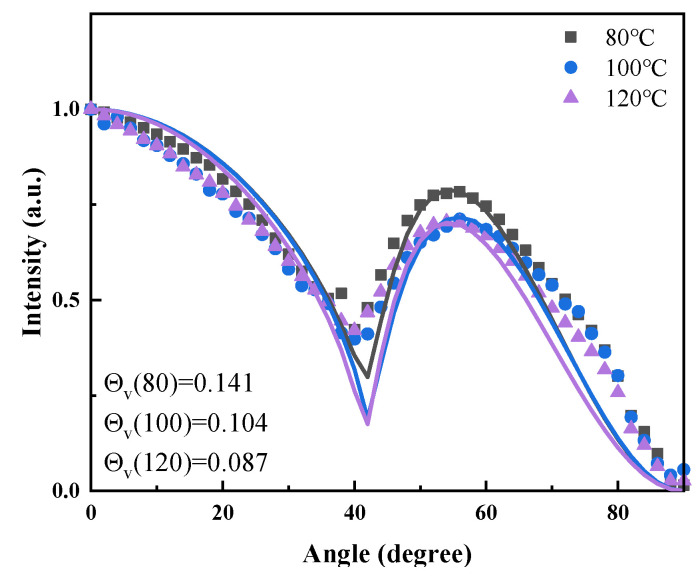
TDM of NPB evaporated on SAM at 80, 100, and 120 °C.

As an example, the angle-dependent fluorescence spectroscopy test and fitted TDM annealing at 80 °C, 100 °C, and 120 °C are shown in Figure 5. Compared to the reference sample, the TDM orientation shifts with varying annealing temperatures. When annealing temperatures are 80 °C, 90 °C, 100 °C, 110 °C, and 120 °C, the vertical dipole moment orientation factors Θ_v_ are 0.141, 0.118, 0.104, 0.095, and 0.069, respectively. The vertical moment orientation factors Θv decrease with increasing annealing temperature. The vertical dipole moment of the NPB molecule without a SAM is 0.112, which is between that at temperatures of 90 °C and 100 °C.

**Figure 6:**
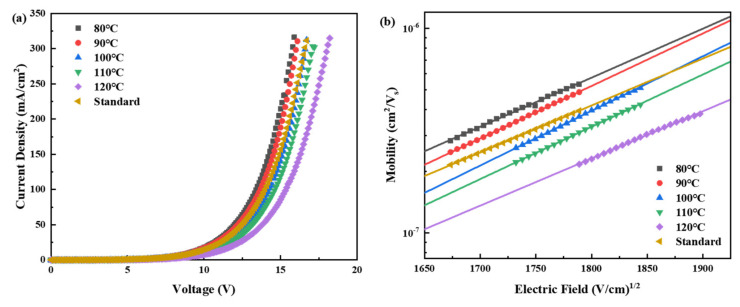
(**a**) J-V curve and (**b**) hole mobility of the HOD with the structure ITO/Me-4PACz/NPB (30 nm)/Alq_3_ (50 nm)/MoO_3_ (15 nm)/Ag (80 nm).

The variation in the annealing treatments of the Me-4PACz film induces changes in the HTL, which in turn significantly affect the injection and transport of charge carriers. Subsequently, a hole-only device with the structure ITO/Me-4PACz/NPB (30 nm)/Alq_3_ (50 nm)/MoO_3_ (15 nm)/Ag (80 nm) was fabricated. Similarly to the OLED, a pure NPB (30 nm) reference device was selected as the “Standard” structure. MoO_3_ served as the electron blocking layer. The current density–voltage characteristics were measured and analyzed, with the corresponding data presented in Figure 6. According to the space-limited-current method (SCLC), when the trap is filled, the current density and voltage satisfy the following relation: $J_{SCLC}=\frac{9}{8}\varepsilon_{0}\varepsilon_{r}\mu \frac{E^{2}}{L}$ [25]. The current density J is influenced by the vacuum and the relative permittivity $\varepsilon_{0}$, $\varepsilon_{r}$, and the carrier mobility μ. The relationship between the carrier mobility μ and the electric field can be described by the Poole–Frenkel formula: $\mu \left(E\right)=\mu_{0}exp\left(\beta \sqrt{E}\right)$, where $\mu_{0}$ represents the mobility of the zero-field charge carrier, and β represents the Poole–Frenkel factor. As shown in Figure 6, under the same voltage conditions, both the current density and hole mobility decrease as the annealing temperature increases. This is consistent with the trend observed in the J-V curves of the OLED devices in Figure 2c, where the current density gradually decreases with increasing annealing temperature at the same voltage.

**Figure 7:**
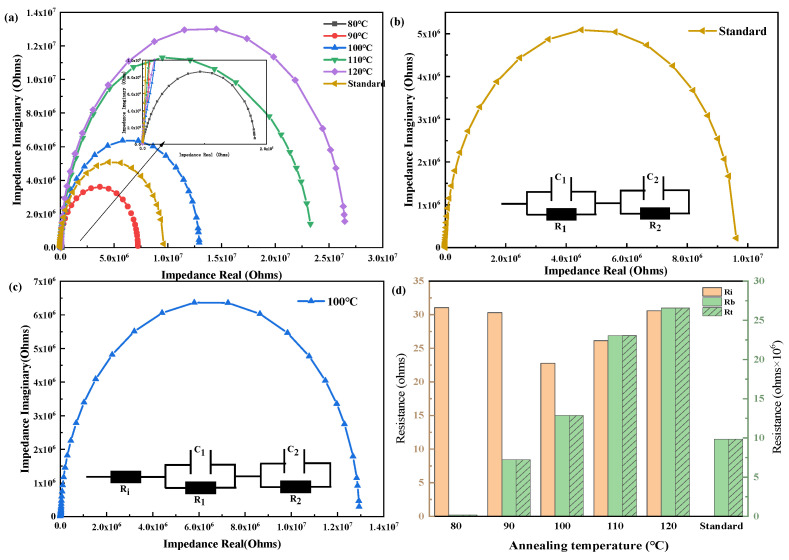
(**a**) Ac impedance spectra of hole-only devices, (**b**) equivalent circuit diagram of the “Standard” HOD (which does not contain Me-4PCz), (**c**) equivalent circuit diagram for annealing at 100 °C, and (**d**) resistance values at different annealing temperatures.

At the same time, electrochemical impedance spectroscopy (EIS) tests were carried out on HODs treated at different annealing temperatures. Figure 7a illustrates the results of the Nyquist curve fitting, with the inset in the figure providing an enlarged view of the portion corresponding to an annealing temperature of 80 °C. Figure 7b displays an equivalent circuit diagram for reference devices without a SAM, which consists of two parallel RC units [26]. The sum of R1 and R2 represent components of the device’s bulk resistance (R_b_). For devices with a SAM, depicted in Figure 7c, the equivalent circuit structure includes two RC units in parallel and one Rs unit in series. The total resistance is the sum of these three components, with Rs representing the interface resistance (R_i_) introduced by the SAM. The emerging interfacial resistance in the equivalent circuit of devices with a SAM illustrates the modifying impact of a SAM between ITO and the HTL NPB. In the case of HODs with added SAMs, the total resistance (R_t_) of the equivalent circuit comprises both interfacial R_i_ and R_b_, with R_b_ playing a dominant role. Furthermore, both the horizontal coordinate and diameter of the Nyquist plot expand as the annealing temperature increases. This trend suggests that the resistance of HODs increases as the percentage of vertically oriented molecules decreases. A statistical graph illustrating R_t_ and R_i_ can be found in Figure 7d. While the R_t_ of the device gradually rises with annealing temperature, R_i_ decreases consistently as the annealing temperature of the SAM ranges from 80 °C to 100 °C. However, as the SAM annealing temperature increases from 100 °C to 120 °C, R_i_ gradually increases. This variation is inconsistent with that in surface roughness.

The performance of the device improved when the optimized SAM was added. The annealing temperature influences the interface modification between ITO and NPB, the TDM of NPB films, and hole mobility. As electron mobility remains constant, the recombination of holes and electrons in the luminescent layer tends to saturate as the annealing temperature rises from 80 °C to 100 °C and subsequently changes gradually as the temperature continues to increase from 100 °C to 120 °C. This suggests that the variation in OLED device performance results from the combined effects of annealing temperature on TDM and carrier mobility.

## 4. Conclusions

This study investigates the enhancement of OLED performance by optimizing HTL with a SAM subjected to various annealing temperatures. The OLED with a SAM annealed at 100 °C achieved the maximum luminous intensity of 32,290 cd/m^2^, more than two times higher than that of the reference device. As the SAM annealing temperature increased from 80 °C to 120 °C, both the vertical TDM in the HTL and the hole mobility in HODs improved. The vertical TDM orientation enhances hole mobility. The analysis of electrochemical impedance spectroscopy and surface morphology suggests that the introduction of a SAM yields interface resistance. The combined effect of changes in TDM and interface morphology optimizes hole mobility in HODs, ultimately leading to improved OLED performance.

## Data Availability

The original contributions presented in this study are included in the article. Further inquiries can be directed to the corresponding authors.
